# Neuromechanical Biomarkers for Robotic Neurorehabilitation

**DOI:** 10.3389/fnbot.2021.742163

**Published:** 2021-10-27

**Authors:** Florencia Garro, Michela Chiappalone, Stefano Buccelli, Lorenzo De Michieli, Marianna Semprini

**Affiliations:** ^1^Rehab Technologies, Istituto Italiano di Tecnologia, Genoa, Italy; ^2^Department of Informatics, Bioengineering, Robotics and Systems Engineering, University of Genoa, Genoa, Italy

**Keywords:** robotic rehabilitation, upper limb rehabilitation, motor control, EMG, EEG, kinematic measurement, stroke, exoskeleton

## Abstract

One of the current challenges for translational rehabilitation research is to develop the strategies to deliver accurate evaluation, prediction, patient selection, and decision-making in the clinical practice. In this regard, the robot-assisted interventions have gained popularity as they can provide the objective and quantifiable assessment of the motor performance by taking the kinematics parameters into the account. Neurophysiological parameters have also been proposed for this purpose due to the novel advances in the non-invasive signal processing techniques. In addition, other parameters linked to the motor learning and brain plasticity occurring during the rehabilitation have been explored, looking for a more holistic rehabilitation approach. However, the majority of the research done in this area is still exploratory. These parameters have shown the capability to become the “biomarkers” that are defined as the quantifiable indicators of the physiological/pathological processes and the responses to the therapeutical interventions. In this view, they could be finally used for enhancing the robot-assisted treatments. While the research on the biomarkers has been growing in the last years, there is a current need for a better comprehension and quantification of the neuromechanical processes involved in the rehabilitation. In particular, there is a lack of operationalization of the potential neuromechanical biomarkers into the clinical algorithms. In this scenario, a new framework called the “Rehabilomics” has been proposed to account for the rehabilitation research that exploits the biomarkers in its design. This study provides an overview of the state-of-the-art of the biomarkers related to the robotic neurorehabilitation, focusing on the translational studies, and underlying the need to create the comprehensive approaches that have the potential to take the research on the biomarkers into the clinical practice. We then summarize some promising biomarkers that are being under investigation in the current literature and provide some examples of their current and/or potential applications in the neurorehabilitation. Finally, we outline the main challenges and future directions in the field, briefly discussing their potential evolution and prospective.

## Introduction

Motor impairment due to neural diseases, such as stroke, is the third most common cause of the global burden of disease according to the WHO following neonatal conditions and heart diseases (WHO, 2019). In 2016, there were 80.1 million prevalent cases and 13.7 million new stroke cases in the world (Johnson et al., 2019). In particular, motor impairment of the upper limb occurs in 73–88% of the first time stroke survivors and in 55–75% of the patients with chronic stroke (Lawrence et al., 2001). The economic impact of this issue represents €60 billion annually only in the European Union, comprising healthcare costs of €27 billion, social care costs of €5 billion, and €16 billion due to the opportunity cost of the informal care by the support system of the patient (family and friends), along with a loss of the productivity costing €12 billion caused by the morbidity or death (Luengo-Fernandez et al., 2020).

Growing efforts have been done to improve the rehabilitation interventions (Frontera et al., 2017; Hayward et al., 2019), which rely on the effective diagnostic of the motor deficit, the accurate evaluation of the recovery or adaptation, and the optimized treatment for the recovery during the chronic stage. For this reason, a wide variety of strategies has been developed for the purpose of the motor restoration (Lin et al., 2019).

**Figure 1:**
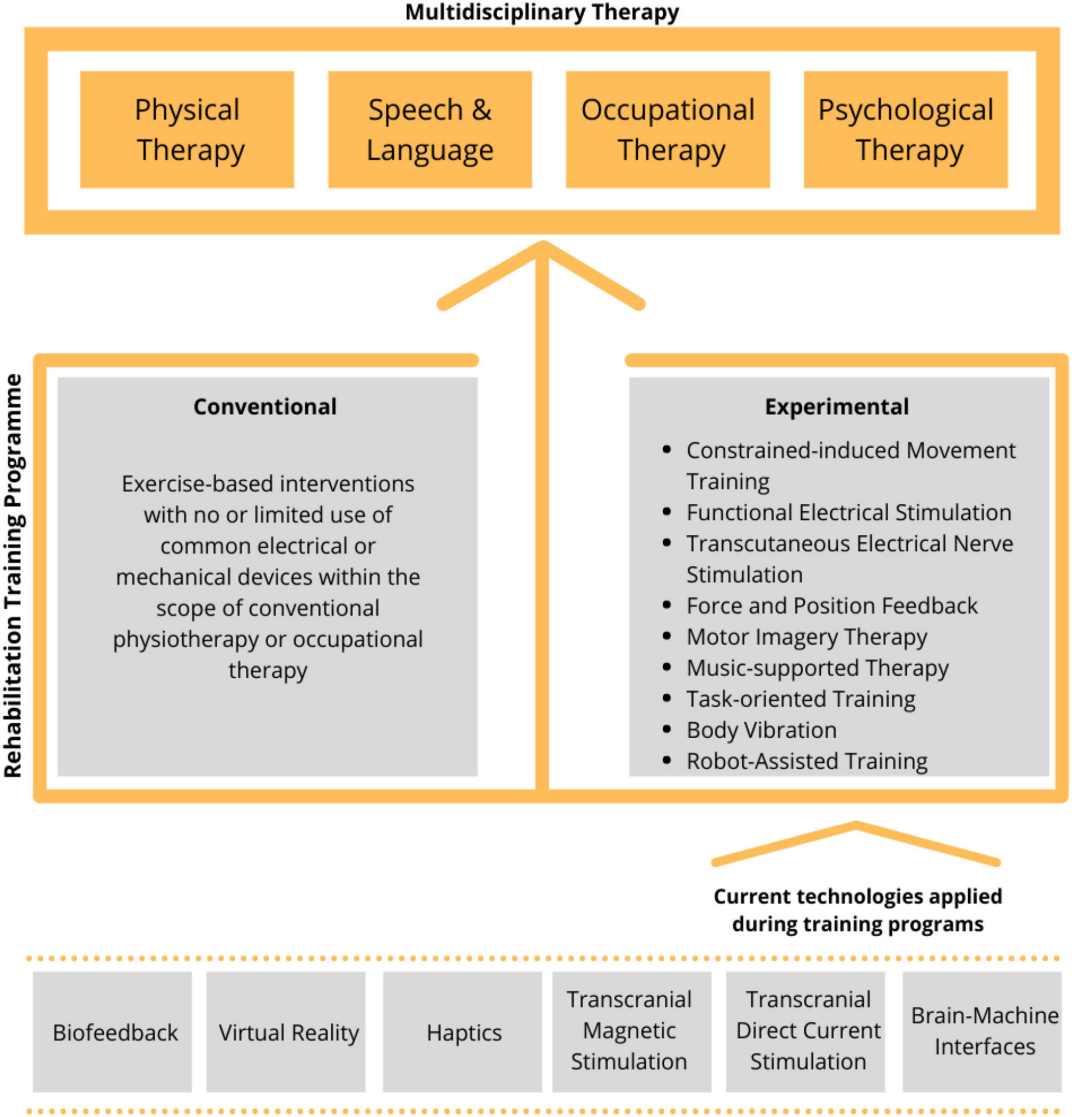
Outline of the current training approaches and technologies used in the rehabilitation. A rehabilitation training program (middle) is used to support the multidisciplinary therapy (top). Rehabilitation training can be either conventional or experimental and the latter being found on one or more available technologies (bottom).

For example, stroke rehabilitation usually involves a rehabilitation training program based on a multidisciplinary approach (including physical, occupational, psychological, and speech therapy), which requires the intervention of many specialists (Figure 1, top).

During the rehabilitation intervention, the training program is continuously tuned and monitored to maximize the functional independence of the patient. These programs aim at promoting the motor learning by stimulating the mechanisms of the brain plasticity, especially during the first 3 months following the brain injury when the probability of the function recovery is greater (Prabhakaran et al., 2008). However, there is solid evidence that the mechanisms of the brain plasticity associated to recovery may continue many years after stroke and the chronic patient can also benefit from the rehabilitation interventions (Irimia et al., 2018).

**Figure 2:**
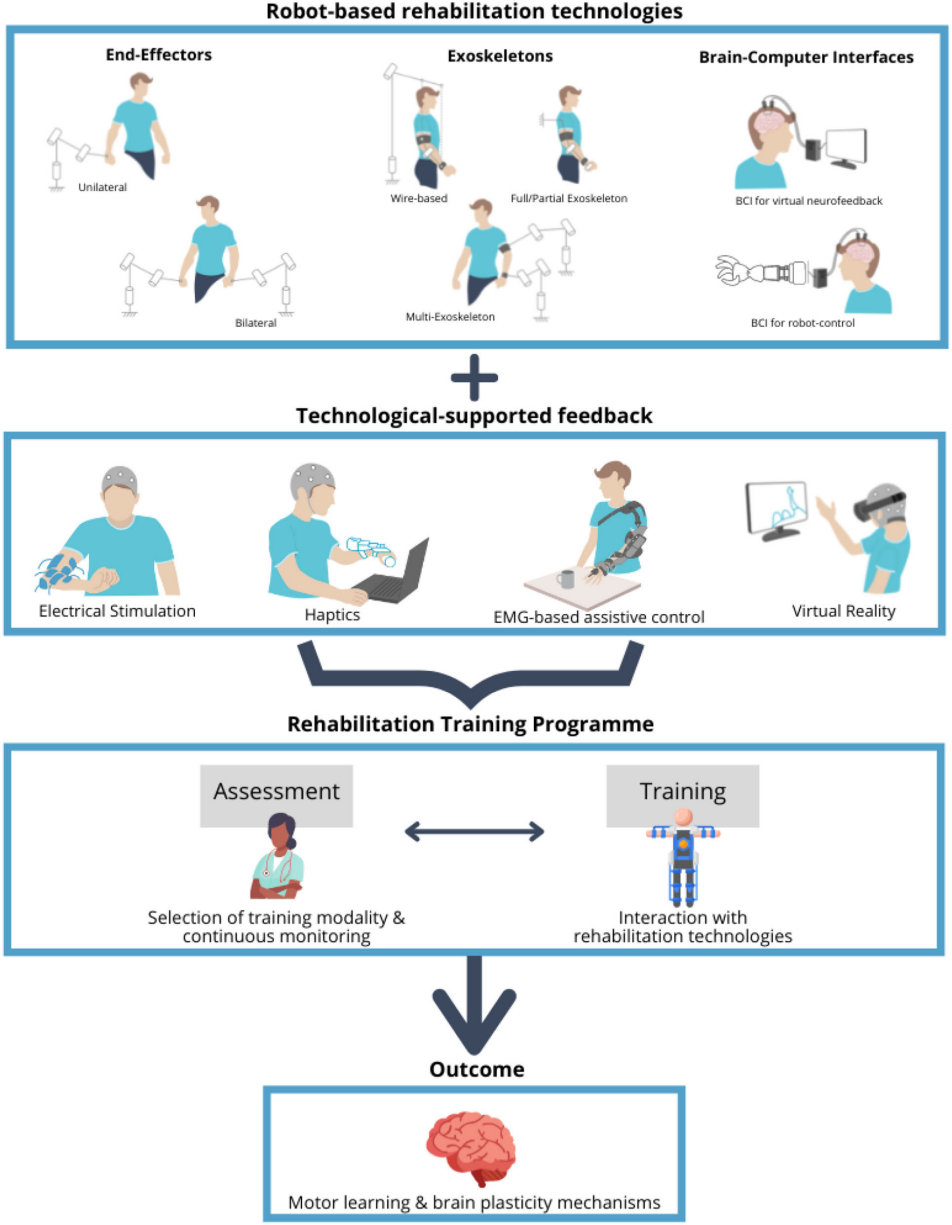
Overview of the robot-based rehabilitation technologies, feedback modalities, and rehabilitation training program. Robot-based rehabilitation technologies (top panel), which include the end-effector robots, exoskeletons, and brain–computer interfaces (BCIs), are used in combination with the feedback modalities (middle panel), ranging from electrical stimulation to haptics, electromyography (EMG)-based assistance, and virtual reality, in order to support the rehabilitation training program (bottom panel). Training program includes the assessment sessions to tune and monitor the specific treatment, aimed at promoting the motor learning by stimulating the mechanisms of the brain plasticity. Schematics in the top panel represent the degrees of freedom of movement for the different types of the end-effector robots and exoskeletons.

The rehabilitation training itself can be either conventional or experimental (Figure 1, middle) (Lin et al., 2019) and the latter supported by one or more available technologies such as robotics, muscle and brain stimulation, and virtual reality (Figure 1, bottom). In particular, in the recent years, robot-mediated therapy has been increasingly used in the rehabilitation to enable the highly adaptive, repetitive, intensive, and quantifiable physical training (Semprini et al., 2018; Iandolo et al., 2019). Robot-based rehabilitation is mainly supported by the end-effector robots, exoskeletons, and brain–computer interfaces (BCIs) (Figure 2, top panel), used in combination with real-time feedback to the patient, which is based on a feedback technology such as electrical stimulation, haptics, electromyography (EMG)-based assistance, and/or virtual reality (Figure 2, middle panel). The combination of these technologies can be used to create a personalized rehabilitation training program (Figure 2, bottom panel). For a comprehensive review on the current robotic technologies applied on the neurorehabilitation see (Nizamis et al., 2021).

## What is a Biomarker and its Relevance for Robot-Assisted Rehabilitation?

Many studies have shown that multidisciplinary robot-assisted training results in an additional reduction of motor impairments in comparison to the traditional rehabilitation approach in the different stages of recovery (Franceschini et al., 2020; Khalid et al., 2021). These effects on motor learning are mainly due to the precise feedback and assistance provided to the patients during practice. It has been demonstrated that not only this can improve the motivation of the patient, engagement, and adherence to the treatment, but also enhance the learning and recovery (Schmidt and Young, 1991; Zhang et al., 2017).

Although there are many studies addressing the clinical benefits of these interventions, the comparison of the clinical effectiveness of the robot-assisted training has had diverse results, with some clinical trials showing that the robot-assisted training did not improve motor function when compared with usual care (Rodgers et al., 2019), thus leading to the controversy in the field.

This has been primarily attributed to the individual clinical factors (age, stroke severity, infarct location, and comorbidities) and the unique profile of the patient (Prabhakaran et al., 2008), which lead to the need of tailoring the treatment and developing the useful parameters to interpret the heterogeneous clinical outcomes (Irimia et al., 2018). In this regard, the robot-assisted interventions provide the therapists with the objective, accurate, and repeatable measurements of the functions of the patient, which allow to objectively follow progress, to evaluate the effectiveness of the different treatments, or to adapt to the specific needs of the patients.

**Figure 3:**
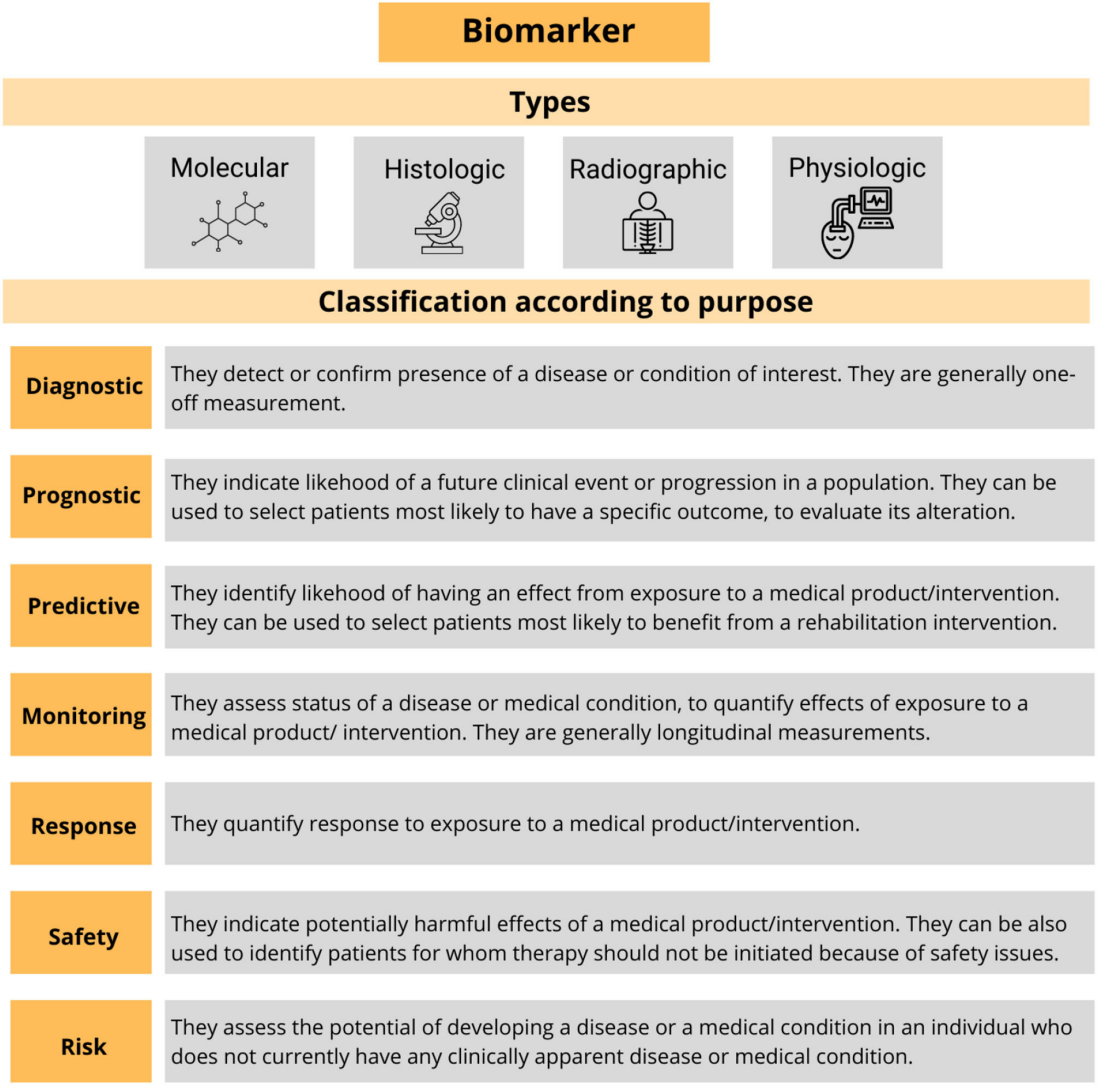
Summary of the types of the biomarkers and their formal classification. Adapted from Biomarkers Definitions Working Group (2001).

These measurements are formally named *biomarkers*. The term refers to a broad subcategory of the medical signs, which are “indicators of the normal biological processes, pathogenic processes, or responses to an exposure or intervention, including therapeutic interventions accurately and reproducibly measured from outside the patient” (Biomarkers Definitions Working Group, 2001). Thus, a biomarker can be molecular, histologic, radiographic, or physiologic and they can be formally classified according to its alleged application (Figure 3). The use of the biomarkers that have been well-characterized and validated across a variety of treatments and populations has become common in the research and in the clinical practice (Mayeux, 2004).

Nevertheless, in many cases, the level of evidence for the validation of the biomarkers does not allow their translation to clinical practice. This is the case of motor rehabilitation, where there is a current need for the objective evaluation and the correct prediction of the outcomes by using the robust biomarkers specific to an intervention. Thus, robot-assisted rehabilitation may help to improve the motor rehabilitation after stroke, traumatic brain injury, and the other neurologic disorders.

For example, the randomized controlled trials comparing the robot-assisted arm training with the other rehabilitation or placebo interventions showed improvement of the activities of daily living, arm function, and arm muscle strength in the post-stroke individuals (Mehrholz et al., 2018). However, the huge variations in terms of intensity, duration, amount of training, type of treatment, characteristics of the participant, and measurements used so far suggest caution in the interpretation of these results (Mehrholz et al., 2018). In this regard, the biomarkers might help to harmonize these results by providing more accurate information and helping to identify the proper respondent at the different technologies, enhancing the stratification of the patients. Nevertheless, the majority of this research is still exploratory: while the literature indicates a growing number of the potential biomarkers and indicators for the several pathologies characterized by the motor impairments, a gold standard rehabilitation-focused biomarker is still lacking at the clinical and preclinical levels (Wagner, 2014).

The growing number of clinical studies evaluating the effects of robotic training on rehabilitation generally relies on the traditional human-administered clinical scales, which often lack of resolution to detect subtle changes in the performance of the patient and can be subjective to the expertise of the physician. Recent studies are indicating that these clinical behavioral biomarkers are less predictive of the motor recovery compared to the neurophysiological biomarkers (Cramer et al., 2007; Quinlan et al., 2018; Lim et al., 2020).

Rehabilitation biomarkers are gradually evolving from simple clinical behavioral metrics based on quantitative scales to brain imaging and neurophysiological measurements (Babrak et al., 2019). There are many studies addressing the relationship between the validated clinical scales and instrumented biomarkers (Zollo et al., 2011; Kim et al., 2016; Connell et al., 2018; Do Tran et al., 2018; Saes et al., 2019; Rech et al., 2020; Riahi et al., 2020; Agrafiotis et al., 2021), but a standardized approach is still missing.

**Figure 4:**
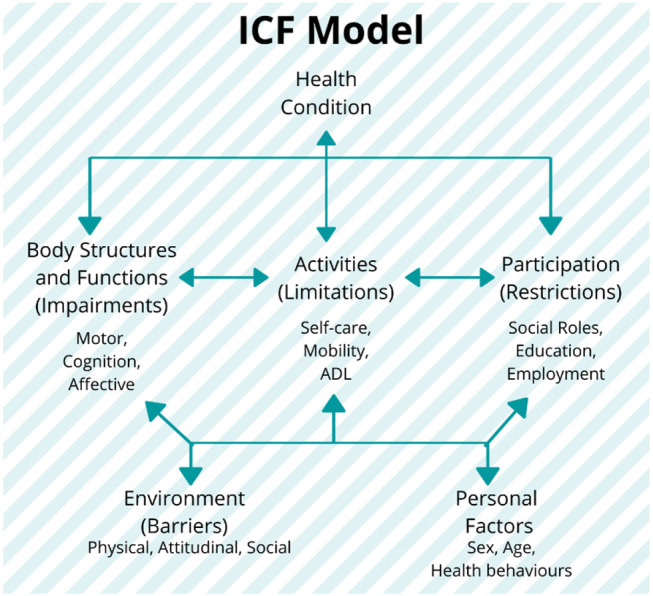
The International Classification of Functioning, Disability, and Health (ICF) model and its components: the model establishes the three levels of human functioning: (1) at the level of body or body part (body structures and functions domain), (2) the whole person (activities domain), and (3) the whole person considered in a social context (participation domain). In this classification, disability implies a certain degree of dysfunction at one or more of these same levels: impairments, activity limitations, and participation restrictions, respectively. It also includes the additional information on the personal and environmental factors (World Health Organization, 2002). Figure is open access courtesy of the National Academies of Sciences (2021) (Trang et al., 2020).

In this regard, efforts like the International Classification of Functioning, Disability, and Health (ICF), proposed by the WHO in 2001 (Stucki et al., 2002; World Health Organization, 2002), have been developed as a standardized framework of assessment, with the purpose of providing an integrated biopsychosocial model to describe the functioning in the rehabilitation (Figure 4). This model describes the health condition as influenced by the several factors related not only to the conditions of body structures and functions as a consequence of the impairment, but also to the repercussions on the activities and social participation of the subjects, which are, in turn, related to both the environmental barriers and personal factors. The ICF model allows for an assessment of the degree of disability regardless of the health condition, etiology of the disease, cultural background, age group, and gender (World Health Organization, 2002).

Thus, this framework introduces the need of a standardized and multidisciplinary approach for the development of measurement that can describe and evaluate the motor rehabilitations focusing on the unique (and multidomain) profile of the patient. Currently, this model has been used as a reference for the clinical practice, but its use in the research is still limited, mostly due to a lack of correlation in the literature between the clinical outcome measures and quantitative parameters such as kinematic and neurophysiological measurements. The categorization of these parameters in accordance with the ICF domains and their connection with clinical scales could provide the additional insights for the selection of the appropriate biomarkers and clinical scales in the assessment of the motor performance (see section Toward Personalized Neurorehabilitation: Adopting the Rehabilomics Approaches in the Robot-Assisted Rehabilitation for the further details).

## Focus on Stroke: Current Biomarkers Related to Motor Recovery

Among the neurological diseases characterized by motor impairments, stroke is one of the most commonly studied. In this context, viable biomarkers of motor recovery have evolved along with brain imaging and neurophysiological technology in the past decades. While brain imaging techniques such as diffusion tensor imaging (DTI), transcranial magnetic stimulation (TMS), functional MRI (fMRI), and conventional structural MRI (sMRI) have been systematically used for establishing the neurologic biomarkers (Buma et al., 2010; Kim and Winstein, 2017), the neurophysiological techniques [such as electroencephalography (EEG) and surface EMG (sEMG)] and kinematic measurements have been explored mainly in the research contexts (Stinear, 2017). Thus, regardless of the evident evolution, there is a shortfall in the high-level evidence for defining the most critical biomarkers of the motor rehabilitation based on the electrophysiology and kinematics measurements (Kim and Winstein, 2017).

In view of the wide variety of the biomarkers under development and their heterogeneity of the applications in the rehabilitation (depending on the neuroimaging method, condition of the patient, training modality, etc.), the following subsections provide an non-exhaustive overview of the biomarkers for the robot-assisted upper limb rehabilitation post-stroke focused on: (1) sEMG, which has been considered a “muscle activation measurement tool” in the past four decades, leading to a wide exploration in neurorehabilitation (Campanini et al., 2020) (Table 1); (2) EEG, which is widely used in the different clinical areas as non-invasive real-time tool to extract the features from the electrical activity of brain and presents high correlation with the various different pathologies (Table 2); and (3) robotic-based kinematic measurements, which have been extensively explored as a potential tool for assessing the motor functions (Table 3).

**Table 1:** List of the electromyography (EMG)-based biomarkers related to the motor rehabilitation focused on stroke.

**1.**	**Muscular Synergies (MSyn)**
	**Definition:** A MSyn is a model that reduces the dimensionality of muscle control, by decomposing the activation of a group of muscles to produce a particular movement (Bizzi and Cheung, 2013; Overduin et al., 2015)
	**Measurement:** MSyn are generally addressed by applying linear decomposition algorithms (PCA, NNMF, and ICA) to extract spatiotemporal, temporal, and spatial features from EMG (Grinyagin et al., 2005)
	**State of the art:** Although MSyn are being widely explored as neuromechanical models for robotic control, there is a current discussion about whether: (1) MSyn have a neural origin, (2) are encoded in the central nervous system, (3) are activated because of task constraints (Severini et al., 2020)
	**Comment on current/potential applications:** Changes in MSyn after post-stroke robot-assisted rehabilitation showed larger improvements in axial-to-proximal muscle synergies with respect to usual care rehabilitation (Lencioni et al., 2021). Measurement of the temporal correlation between the recruitment of MSyn of paretic and healthy muscles on post-stroke survivors shows correlation of these synergy-based measures with clinical scores, and is proposed as a physiological biomarker of motor function and recovery in stroke, called Functional Synergy Recruitment Index (Irastorza-Landa et al., 2021)
**2.**	**Intermuscular coherence (IMC)**
	**Definition:** IMC consists in identifying correlated patterns of EMG to analyze muscle coordination during a specific task (Giszter, 2015). It has been proposed that it evidences the shared frequencies at which a group of muscles are modulated by common neural drive (Farina et al., 2016)
	**Measurement:** IMC is measured by means of time-domain correlation and spectral coherence analysis to characterize muscle binding
	**State of the art:** As with MSyn, it is often uncertain whether correlated muscle activity reflects their neural binding or just the constraints imposed by the task (Laine and Valero-Cuevas, 2017)
	**Comment on current/potential applications:** No current works applying IMC to robotic-based rehabilitation directly were found However, the exploration of ICM in both healthy and stroke subjects have shown that a different number of muscle networks is required for the activation of the upper arm and elbow muscles, suggesting a simplification of the functional motor control scheme in post-stroke subjects (Houston et al., 2020)
**3.**	**EMG Time and Frequency Domain Features**
	**Definition:** Time domain features are related to transient EMG properties which are calculated based on raw EMG time series, while frequency domain features are related to the EMG properties which are calculated based on the power spectral density (PSD) of the EMG (Phinyomark et al., 2012; Nazmi et al., 2016)
	**Measurement:** For a detailed description of each feature equation, see (Phinyomark et al., 2012; Nazmi et al., 2016)
	**State of the art:** EMG features have been widely explored in robotic control and assessment of rehabilitation following brain injury in the past decade (Leonardis et al., 2015; Cahyadi et al., 2018a; Majid et al., 2018). While novel techniques are continuously being developed (Pancholi et al., 2019), there is still a lack of consensus in both nomenclature and computation of these features, which is preventing from their implementation as a clinically relevant biomarkers, or as standardized control parameters for robotic systems. Current efforts in building consensus about EMG techniques and terminology are homogenizing the execution and communication of EMG studies across different disciplines (McManus et al., 2021). In addition, hybrid time-frequency features are proposed to overcome the limitation of time features, which relies in stationary properties of the EMG signal. These features are less applied due to computation costs, and are on time-frequency methods such as Discrete Wavelet Transform and Wavelet Packet Transform (Phinyomark et al., 2012; Nazmi et al., 2016)
	**Comment on current/potential applications:** Currently, EMG features are being used to the enhancement of robot-assisted upper limb rehabilitation platforms, by means of using the subject's intentions to generate proper feedback for the robotic system (Cahyadi et al., 2018b; Bouteraa et al., 2020; Khairuddin et al., 2021). In particular, due to their relative low computational cost, their potential combination with machine learning algorithms and other technologies such as virtual reality (Meng et al., 2019) could be the key to develop dynamic rehabilitation devices that can boost the personalization of motor training (Abdallah et al., 2017; Arteaga et al., 2020; Samuel et al., 2021)
**4.**	**Motor Unit Decomposition based on HD-sEMG**
	**Definition:** The decomposition of high-density (HD) sEMG has been recently developed as a technique to decode descending neural drive out of the timing of motoneurons discharge (Farina et al., 2017), which can allegedly be more sensitive to decode the user intent of movement than traditional sEMG techniques
	**Measurement:** HD-sEMG is achieved by embedding EMG electrodes into 2D arrays, increasing the detection volume without compromising the bandwidth of the recorded sEMG signals, and then algebraically combining them to create spatial maps that are sensible to the propagation of the motor unit action potential (Farina and Holobar, 2014)
	**State of the art:** Currently there are few publications regarding potential application to robot-assisted rehabilitation, as this technique has begun to be explored more in recent years. In particular, the analysis of intramuscular motor unit coherence has been proposed as a potential measurement for gait rehabilitation (Úbeda et al., 2019). Non-invasive approaches have also been proposed, applying PCA techniques to HD-sEMG to characterize hand movements during grasping tasks (Tanzarella et al., 2020), and paretic leg during fatiguing contractions for potential correlations with post-stroke motor behavior and gait performance (Negro et al., 2020)
	**Comment on current/potential applications:** There is a growing interest in HD-sEMG decomposition as a way to characterize neural control by modeling the state of the human neuromuscular system. This would help tackling some of the most urgent health challenges, including motor dysfunctions (Holobar and Farina, 2021). Among the main challenges for developing this technique, it is worth mentioning the assessment of inter-operator reliability of identification of motor unit spike trains from HD-sEMG (Hug et al., 2021) and complexity introduced by task constraints and the correct interpretation of the task-specific modulation (i.e., isometric vs. dynamic tasks), along with the challenges involved in the signal processing, such as the dimensionality reduction of HD-sEMG signals (Holobar and Farina, 2021)
**5.**	**Muscle Fatigue**
	**Definition:** Muscle fatigue does not constitute a direct measurement of motor function, because it is formally defined as an exercise-induced reduction in muscle performance (Maffiuletti and Bendahan, 2009). Thus, it provides a functional parameter for the assessment of neuromuscular and metabolic mechanisms that underlie fatigue, not motor function. However, muscle fatigue does influence performance in motor impairment, and it has been explored as a complementary biomarker for rehabilitation, for quantifying the effects of fatigue in the performance of different interventions, such as virtual reality (Montoya et al., 2020). Muscle fatigue has been widely studied in robot-based rehabilitation to address the phenomenon of fatigue compensation during rehabilitation, which can lead patients to recruit trunk and shoulder during arm movements, causing an undesirable rehabilitation and risks of injury (Huang et al., 2019)
	**Measurement:** Muscle fatigue is mainly assessed through time and/or frequency-domain features of the EMG signal, such as the mean and the median frequency. These time-frequency based features are usually fed to machine learning algorithms (like K-nearest neighbor, naïve Bayes and genetic algorithm based support vector machine) in order to recognize the onset of muscle fatigue (Venugopal et al., 2014). Different methods for selecting relevant features have been proposed to optimize the classification (Karthick et al., 2018; Wang J. et al., 2020; Makaram et al., 2021)
	**State of the art:** Muscle fatigue is a common factor that influences recovery and motor performance. It has been widely investigated in the rehabilitation area, aiming at creating adaptive rehabilitation systems that be taken into account to make real-time adjustment to the interventions. In particular in stroke rehabilitation, the effects of muscular fatigue have been explored in patients with post-stroke spasticity which present abnormal antagonistic muscle co-activation patterns, because there exist a significant influence of muscle fatigue on the coupling of antagonistic muscles (Wang L.-J. et al., 2020)
	**Comment on current/potential applications:** The exploration of potential adaptive robotic system for rehabilitation using muscle fatigue as a trigger has been tested for improve engagement and performance (Meyer-Rachner et al., 2017; Mugnosso et al., 2018; Huang et al., 2019; Kanal et al., 2019). Novel methods for fatigue detection are continuously being developed, boosted by machine learning algorithms and wearables EMG sensors (Mugnosso et al., 2017; Papakostas et al., 2019; Wang W. et al., 2020; Liu et al., 2021)
**6.**	**Motor Unit Number Index (MUNIX)**
	**Definition:** MUNIX is an indirect indicator of the number of functional lower motor neurons innervating a muscle (Nandedkar et al., 2004; Neuwirth et al., 2016)
	**Measurement:** MUNIX is based on a mathematical model described by Nandedkar et al. (2004), in which compound muscle action potentials (CMAPs) and electromyographic (EMG) interference patterns are used to obtain a rapid estimation (3–5 min per muscle) of motor unit numbers (Neuwirth et al., 2010)
	**State of the art:** It is mostly used as indicator of disease progression in motor unit diseases like ALS (Fatehi et al., 2018)
	**Comment on current/potential applications:** No current works directly applying MUNIX to robotic-based rehabilitation were found. Exploration of MUNIX in stroke survivors to assess spinal motoneuron loss in paretic muscles has shown a significant decrease in MUNIX values in the paretic muscles, as compared with the contralateral muscles (Li et al., 2011)

**Table 2:** List of the electroencephalography (EEG)-based biomarkers related to the motor rehabilitation focused on stroke.

**1**.	**Functional Connectivity (FC)**
	**Definition:** FC is a widely used technique for mapping the functional organization of the brain, by measuring the temporal correlation of the activation of different brain areas at rest, using fMRI and EEG techniques (Carter et al., 2012; Siegel et al., 2016)
	**Measurement:** FC can be computed from EEG signals applying connectivity techniques. There exist many approaches for calculating FC, the most used ones are based on linear coherence (Bowyer, 2016). Generalized partial directed coherence (GPDC) has also been broadly used due to its performance and noise robustness (Fasoula et al., 2013). Graph theory metrics are often used in FC studies, to explore network properties (Bullmore and Sporns, 2009). Other methods, such as those based on Granger causality theory, allow not only to show the information flow from different brain regions, but also its directionality (Friston, 2011)
	**State of the art:** There is a growing interest in using changes in FC to assess rehabilitation training effects, but few studies are actually using it to characterize or predict outcomes (Yuan et al., 2021). In particular, potential biomarkers for stroke rehabilitation could arise from the exploration of altered functional interactions that are highly correlated with motor behavioral deficits and post-stroke recovery (Siegel et al., 2016; Caliandro et al., 2017; Wang et al., 2019). Moreover, there is the possibility of combining neuroimaging modalities to enhance the power of FC to investigate brain recovery mechanisms, which is being poorly explored (Yuan et al., 2021)
	**Comment on current/potential applications:** Topological properties of neural networks have been explored as potential biomarkers for post-stroke rehabilitation, in particular resting state EEG parameters such as small world organization (Caliandro et al., 2017; Vecchio et al., 2019), debiased weighted Phase LagIndex (dwPLI) (Issa et al., 2019) and network connectivity average mean degrees (E-PDC) (Eldeeb et al., 2019). Graph theory indexes of brain segregation like modularity and transitivity have also been proposed as biomarkers of motor learning (Miraglia et al., 2018). There are several indexes derived from FC under exploration for their potential application in robot-assisted post stroke interventions, such as the inter-hemispheric strength index (Pellegrino et al., 2012; Pichiorri et al., 2018; Ondobaka et al., 2019). In addition, other neuroimaging techniques such as fMRI has been used for the same purposes (Mohanty et al., 2018), exploring its correlation with EEG to assess stroke recovery from BCI training for upper limb rehabilitation (Yuan et al., 2021)
**2.**	**Cortico-muscular Coherence (CMC)**
	**Definition:** CMC is a well-known approach to assess the synchronization between brain and muscle activity. It is associated to functional connections within the corticospinal pathways, between motor cortex and muscles during movement execution (Liu et al., 2019a)
	**Measurement:** Coherence is defined as the linear relationship between two signals. While there exist many approaches to calculate CMC, it is commonly defined as an extension of Pearson correlation coefficients in the frequency domain (Mima and Hallett, 1999). CMC has been explored using different neuroimaging techniques, namely MEG and EEG, but can also be computed by using EEG, sEMG and electrocorticography (Gerloff et al., 2006). Other methods such as mutual information and transfer entropy have also been explored to overcome the limitations of linear methods and to characterize non-linear correlations (Liang et al., 2020)
	**State of the art:** Currently, the study of CMC is mainly focused on how different brain areas control and modulate the activation of muscles, how the feedback from the muscles is received and processed (Sinha et al., 2020; Ibáñez et al., 2021), and how CMC can be altered due to different conditions (in particular, its modulation by fatigue (Martínez-Aguilar and Gutiérrez, 2019; dos Santos et al., 2020; Wang L. et al., 2020; Padalino et al., 2021). Current literature has established CMC as a biomarker of neurophysiology in healthy subjects (Franco-Alvarenga et al., 2019; Liu et al., 2019b) and sport conditions (Ushiyama et al., 2010). However, the complexity of the interactions within neural and muscle systems creates high inter and intra-subject variability, and it is highly dependent on research conditions. This, among other factors such as age correlation, is preventing the application of CMC as a clinically reliable measurement of motor function (Liu et al., 2019a)
	**Comment on current/potential applications:** The current application of CMC is mostly limited to characterize its changes under different experimental settings, and across conditions, such as stroke (Belardinelli et al., 2017; Krauth et al., 2019), ALS (Proudfoot et al., 2018), and multiple sclerosis (Padalino et al., 2021). In particular, the exploration of CMC for driving brain-computer interface-based neurorehabilitation has been proposed, by using correlation between band-limited power time-courses (CBPT) associated with EEG and EMG(Chowdhury et al., 2019)
**3.**	**β-band event-related desynchronization and synchronization**
	**Definition:** β-band event-related desynchronization (β-ERD) and synchronization (β-ERS) in primary motor cortex (M1)are transitory oscillations in brain activity that reflect the preparation, execution and cessation of movement (Neuper and Pfurtscheller, 2001). In particular,β-ERD is associated with motor preparation, execution and motor imagery (MI), and it indicates the onset of movement in the contralateral postcentral gyrus, propagating to the bilateral sensorimotor cortices (Takemi et al., 2013). β-ERS (commonly named post-movement beta rebound—PMBR) has been correlated with the deactivation of the motor cortex due to an increase of intracortical inhibition. It peaks between 500 and 1,000 ms after the termination of movement, and continues for circa 1 s (Pfurtscheller and Lopes da Silva, 1999)
	**Measurement:** β-ERD and β-ERS are transient events in the spontaneous brain rhythmic activity corresponding to α and β bands (<35 Hz) (Neuper and Pfurtscheller, 2001). Their computation is mainly based in time-frequency analysis of the EEG in the region of interest (ROI) related to motor modulation
	**State of the art:** β-ERD and β-ERS are ones of the most explored EEG features in motor control, namely in the assessment of motor imagery (Rimbert et al., 2017) and motor inhibition (Heinrichs-Graham et al., 2017). In particular, it has been shown that β oscillations can reflect the motor recovery in upper limbs after stroke (Tang et al., 2020). These features have shown high test-retest and intra-individual reliability (Espenhahn et al., 2017), and it has been indicated that their magnitude is not affected by movement features such as length and velocity (Tatti et al., 2019)
	**Comment on current/potential applications:** β-ERD and β-ERS has been widely exploited for motor imagery assessment, both in rehabilitation interventions (Gandolfi et al., 2018; Norman et al., 2018) and device control (Tariq et al., 2018; Huang, 2020). In particular, PMBR has been referred as a potential biomarker in stroke recovery, by predicting the response to motor training and future motor performance after 24 h of the training sessions in chronic stroke patients (Espenhahn et al., 2020)
**4.**	**EEG topographies or EEG microstates**
	**Definition:** EEG topographies (or microstates) are representations of spontaneous brain activity during resting state that characterize a specific brain state by periods of coherent and synchronized neural activation (Pirondini et al., 2017)
	**Measurement:** There exist different methods to compute dominant topographies based on EEG recordings. In particular, singular value decomposition (SVD) has been recently used for the application of EEG topographies to stroke assessment (Pirondini et al., 2020)
	**State of the art:** Typical topographies of 50–150 msec of duration have been persistently observed in healthy subjects (Van de Ville et al., 2010), and have been correlated to subject-specific characterization of motor control (Pirondini et al., 2017). This shows that EEG topographies could be a robust biomarker for diagnostic and prognostic of motor outcomes
	**Comment on current/potential applications:** There are some recent studies proposing their application to the assessment of stroke patients (Pirondini et al., 2020), but their use in clinical settings is still unexplored
**5.**	**Brain Symmetry Index (BSI)**
	**Definition:** BSI is one of the most explored EEG-derived index for stroke assessment (Xin et al., 2012). It quantifies the inter-hemispheric asymmetry by comparing their power spectra
	**Measurement:** BSI measures the inter-hemispheric EEG power asymmetry, by comparing all EEG-relevant frequency bands, thus it is not specific to a particular band power (Van Putten and Tavy, 2004). There exist several formulas to compute BSI, like pairwise-derived Brain Symmetry Index (Fanciullacci et al., 2017), and revised Brain Symmetry Index (rBSI) (van Putten, 2007)
	**State of the art:** BSI is currently being used in research mainly for stroke prognosis (Agius Anastasi et al., 2017). It has been shown that BSI is correlated with the neurological status and with the level of motor recovery in the acute post-stroke phase (Finnigan and van Putten, 2013)
	**Comment on current/potential applications:** BSI has been evaluated during a robot-assisted intervention, supporting the evidence that a BSI reduction is associated with higher motor recovery (Miehlbradt et al., 2019)
**6.**	**Laterality Coefficient (LC)**
	**Definition:** LC is an index that represents the degree of asymmetries of the ERD patterns between brain hemispheres, usually calculated in the beta and SMR frequency bands. It is used to explore the altered brain activity patterns affected by a condition or an intervention (Sebastian-Romagosa et al., 2020)
	**Measurement:** LC parameter is usually calculated as a ratio between the ERD/ERS in the ROI and frequency band of interest, during the experimental tasks (Sebastian-Romagosa et al., 2020)
	**State of the art:** Many studies use LC index in different motor alterations as a quantitative biomarker for assessments of rehabilitation therapy outcomes, including those using BCI and robotic support. LC is a well-known EEG parameter, and it is often reported in clinical studies as complementary information to clinical scales assessments (Sebastián-Romagosa et al., 2019)
	**Comment on current/potential applications:** LC is being used as a relevant parameter to evaluate new technology-based approaches for stroke rehabilitation (Sebastian-Romagosa et al., 2020), such as combined action observation- and motor imagery-based using BCI (Yuan et al., 2020; Rungsirisilp and Wongsawat, 2021) (not limited to EEG-based assessments; Yuan et al., 2020), functional electrical stimulation (Chen et al., 2021), and TDCS (Ang et al., 2015). Following the current trend of multidisciplinary evaluation of biomarkers, LC has also been included as part of a multidomain instrumental evaluation of post-stroke chronic patients, coupled with standard clinical assessments (Belfatto et al., 2018)
**7.**	**Powerband Ratios (PowRa)**
	**Definition:** Power band ratios are qEEG parameters that indicate the relationship between different frequencies present in the EEG, namely: (1) **Power Ratio Index (PRI)**, which is the relationship between slow and fast frequencies. A high value of PRI implies the presence of high power in slower frequencies, which are associated with poor motor performance and poor prognosis (Mane et al., 2019); and **frequency bands ratios**, which are: (a) Delta Alpha Ratio (DAR), (b) Theta Beta Ratio (TBR), (c) Theta Alpha Ratio (TAR), (d). Theta Beta Alpha Ratio (TBAR)
	**Measurement:** PowRa are calculated by using the absolute band power in the frequency bands of interest (Delta, Theta, Alpha, Beta) obtained from their power spectral density, and computing the ratio between them. For instance, PRI is determined as (δ + θ)/(α + β)(Mane et al., 2019)
	**State of the art:** Very limited chronic stroke rehabilitation studies evaluate the prognostic and monitory value of these qEEG indexes for robot-assisted rehabilitation (Trujillo et al., 2017). Their current use is mainly exploratory, although the few evidence about its correlation with clinical scales shows promising correlation with motor recovery, which should be further addressed
	**Comment on current/potential applications:** Previous studies have investigated the relationship between different PowRa and clinical scales in post-stroke patients, looking for intervention-specific biomarkers. However, PowRa are still exploratory, except from TBR that it is currently the only EEG-based index which has been recently validated as a biomarker for Attention-deficit/hyperactivity disorder (ADHD) (Arns et al., 2013) and it is being used as a rehabilitation index for neurofeedback (Kerson et al., 2019)
**8.**	**Sensorimotor Rhythm (SMR)**
	**Definition:** SMR are brain rhythms associated with motor output, which are localized in the motor and somatosensory cortex between 7 and 11 Hz (Mu SMR) and 12-30 Hz (Beta SMR) (Pfurtscheller et al., 1997). In normal movement, Mu rhythms are desynchronized with movement planification and execution, followed by an increase of contralateral Beta SMR, and finally a synchronization of Mu and Beta SMR after movement completion (Pineda, 2005)
	**Measurement:** SMR are mainly calculated by applying spectral analyses based on Fourier transforms to estimate the absolute spectral power in the EEG frequency bands of interest
	**State of the art:** SMR is a well-demonstrated phenomenon, and its voluntary modulation in order to trigger neuroplasticity phenomena has been used to develop two main strategies for motor rehabilitation for stroke patients: motor imagery (Irimia et al., 2016) and attempted movement-based approaches (Remsik et al., 2019) for BCI-based interventions. It has also been broadly explored in neurofeedback for disorders like ADHD, in which many different therapeutic approaches have been discussed (Jeunet et al., 2019)
	**Comment on current/potential applications:** While studies addressing SMR-based interventions are promising, it is still necessary to investigate open issues like the correlation between clinical improvement and neuroplasticity phenomena, the influence of the placebo effect and the impact of the training procedure used In particular, for stroke applications it has been highlighted the need to support the efficiency of BCI/neurofeedback techniques with large clinical studies, and the implementation of appropriate BCI/neurofeedback protocol designs, optimizing the signal processing, the duration and number of sessions, the transfer/generalization methods, among others (Ramos-Murguialday et al., 2013; Arns et al., 2017)

**Table 3:** Kinematic-based biomarkers related to the motor rehabilitation focused on stroke.

**Biomarker**	**Definition**	**Type of Measurement**
Success rate/performance index	Number of accomplished targets divided by the total amount of target	Efficacy
Active Movement Index (AMI)	AMI is related to a robot score (obtained by the patient during the task by active movement), and the theoretical score if the patient was able to complete the tasks by his own voluntary movement	
Number of movements onset	Number of times that the velocity curve exceeded a percentage of peak velocity at least once after the movement onset	
Number of movements ends	Number of times that the velocity curve dropped below a percentage of peak velocity after movement offset	
Task/Movement time	Elapsed time from movement onset to the end of the task or movement	Efficiency
Distance traveled	Distance encompassed from onset to end of a movement or task	
(Normalized) Path Length Ratio	Relationship between the distance between the patient's path and the shortest possible distance between movement onset and end	
Independence	Measurement of the ratio between the x and y axes, in circle tasks. It indicates the degree of circularity of the movement	
Trajectory error variability	Description of the angle between the force vector recorded by robot and the theoretic direction of movement across the trajectory	Precision
Mean velocity variability	Difference among the velocity profile of the participant's reaching trajectory and the ideal velocity profile for each movement	
Variable error	Standard deviation of the endpoint error within multiple repetitions of the movement or task.	
Endpoint error	Difference between actual and target position at end of movement. It measures the amount of deviation of the patient's hand from the desired trajectory	Accuracy
Trajectory error/Movement accuracy	Difference between ideal and real trajectory between movement onset and end	
Axes ratio	The ratio of the axes of the best-fitting ellipse during circle drawing	
Correlation to reference shape/Shape accuracy	Quantification of the ability to draw a square or a circle posted on a visual interface	
Initial movement direction error	Indicates the distance between ideal and real trajectory after movement onset	Movement planning
Time to peak velocity	Calculates the time for reaching the peak velocity, relative to the duration of the movement	
Reaction time	Calculates the time between go signal and actual starting of the movement	
Normalized mean velocity	It indicates the total translation over total movement duration	Smoothens
Normalized Jerk	The jerk metric indicates the rate of change of acceleration in a movement	
Number of Velocity peaks	Indicates the number of peaks above a threshold in the velocity profile during the trajectory	
Number of sub movements	They characterize the sequence of sub movements that compose the arm movement	
Duration of sub movements		
Frequency of sub movements		
Shape of sub movements		
Amplitude of sub movements		
Overlap of sub movements		
Normalized dimensionless jerk	Third time-derivative of position between movement onset and end normalized with respect to movement duration	
Spectral arc length	Length of the spectral trajectory of the velocity profile between movement onset and end	
Movement arrest period ratio	It is the proportion of time that movement speed exceeds a given percentage of peak speed	
Elbow flexion extension angle	Establish the range of the elbow flexion/extension angle during movement	Spatial posture
Shoulder flexion extension angle	Establish the range of the shoulder flexion/extension angle during movement	
Trunk displacement	It is the distance covered by the trunk during movement	
Shoulder abduction/adduction angle	Establish the range of the shoulder abduction/adduction angle during movement	
Elbow Peak Velocity	It is the highest value of the elbow flexion/extension velocity profile during movement	Temporal posture
Trunk movement time	It is the elapsed time between trunk movement onset and end	
Trunk Peak Velocity	It is the highest value of the velocity profile of the trunk between movement onset and end	
Shoulder and elbow correlation	Maximum value of the cross-correlation between the shoulder and elbow time-angle profiles	
Time to peak elbow extension angle	It computes the time to reach peak elbow extension angle, relative to the duration of the movement	
Normalized reaching area	It establish the maximum reachable position during a movement or task divided by the length of the patient's arm	Workspace
Mean velocity error	It is the mean value of the distance between the ideal velocity profile and real velocity	Speed
Peak velocity	It describes the highest value of the velocity profile during movement	
Postural hand speed	The mean hand speed for a specific time windows after target onset	

While there exists a wide variety of the kinematic parameters used to describe the temporal and spatial features of the endpoint or joint movement (such as the position, velocity, movement time, or the execution of a task or action), systematic reviews on the kinematic assessments show that these parameters are poorly standardized and the unbiased clinimetrics is rarely addressed (Schwarz et al., 2019).

Due to the great number of biomarkers in this category and their large variability across the literature in terms of the nomenclature and level of evidence, examples in Table 3 are presented according to the guidelines introduced in Schwarz et al. (2019), in which the clinically relevant kinematic measurements for the upper limb after stroke were selected from a large database according to their available clinimetric evidence and clustered according to their presumed physiological interpretation for both the three-dimensional (3D) and two-dimensional (2D) tasks. With respect to the previous efforts in standardization and the expertise of the authors, this classification considers the following categories:

1. **Efficacy:** Indication if the task or the objective was successfully achieved or not.
2. **Efficiency:** Quantification of the performance of a task.
3. **Precision:** Description of the variability of performance of the goal-directed movements.
4. **Accuracy:** Quantification of error of the performed movements compared with an optimal movement.
5. **Smoothness:** Deviation of the velocity profile from an optimal profile.
6. **Spatial posture:** Position-related aspects of the joints.
7. **Temporal posture:** Time-related aspects of the joints.
8. **Workspace:** Description of the reachable area or volume with a specific joint.
9. **Speed:** Velocity of the performance of the movements.

## Toward Personalized Neurorehabilitation: Adopting the Rehabilomics Approaches in the Robot-Assisted Rehabilitation

The idea of the state-of-the-art biomarker platforms and the technologies focused on rehabilitation have led to the concept of the “Rehabilomics” (Wagner, 2010), i.e., a transdisciplinary evaluation of the biomarkers to understand the rehabilitation-relevant phenotypes related to biology, function, prognosis, treatment, and recovery for the patients with disabilities (Wagner, 2010).

**Figure 5:**
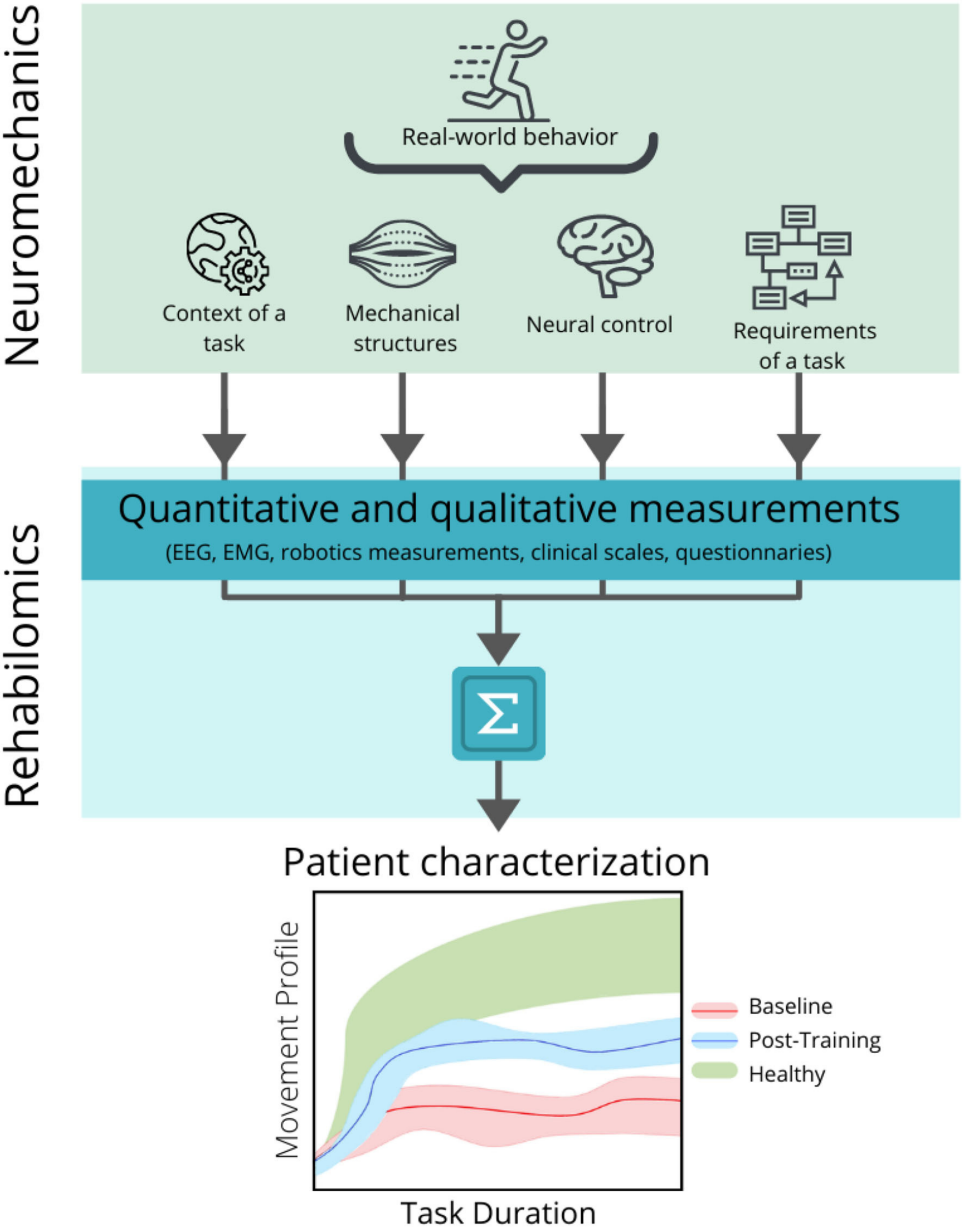
Relationship between the neuromechanical models and the Rehabilomics approach in the development of the motor-related biomarkers. Neuromechanics addresses the real-world behavior by considering the interaction between the context of the motor task, the mechanical structures of the body that are activated to produce the movement, the neural control necessary to produce and modulate the movement, and the specific requirements of the task (top panel). These parameters can be converted into quantitative and qualitative measurements by applying the recording techniques (such as electroencephalography, electromyography (EMG), kinematic measurements, validated clinical scales, and questionnaires) and can be combined to create a personalized profile of the patient (middle panel), in order to assess and predict the motor outcomes related to a specific intervention (bottom panel), before (bottom panel, baseline band in red) and after (bottom panel, post-training band in blue) the rehabilitation, and compare it with a normative band (bottom panel, healthy band in green).

In this context, the development of the biomarkers based on the models of the motor control mechanisms needs to take into account how the real-world behavior emerges from the interaction between the neural, biomechanical, and environmental dynamics, in order to understand the healthy functions, disability, and rehabilitation progress. This perspective is the main purpose of the studies of the neuromechanics (Nishikawa et al., 2007; Valero-Cuevas, 2016), which aims at modeling the healthy movement and studying how these patterns change in the motor deficits, mainly for the robotic design and control (Pham et al., 2014; Szczecinski et al., 2017; Kühn et al., 2018). The research on the biomarker has been mainly focused in a physiological perspective and there is a need for the methodological approaches based on the neuromechanical assessments. In this scenario, the Rehabilomics can provide the new tools to better understand the motor rehabilitation from a multidisciplinary perspective (Figure 5).

Since the Rehabilomics has been primarily focused on the proteomics, genomics and metabolomics (Wagner and Zitelli, 2013; Skriver et al., 2014; Wagner, 2017; Wagner and Kumar, 2019), kinematics measures, and neuroimaging and electrophysiological recordings, they have also been widely explored as the potential biomarkers in the field of the robot-assisted neurorehabilitation (Philips et al., 2017; Belfatto et al., 2018; Pirondini et al., 2018; Krauth et al., 2019; Mane et al., 2019; Irastorza-Landa et al., 2021). In particular, the kinematics and electrophysiological indicators can be exploited as biomarkers, mainly because they are non-invasive and portable techniques, suitable for measuring the activity in both the acute and chronic phases.

**Figure 6:**
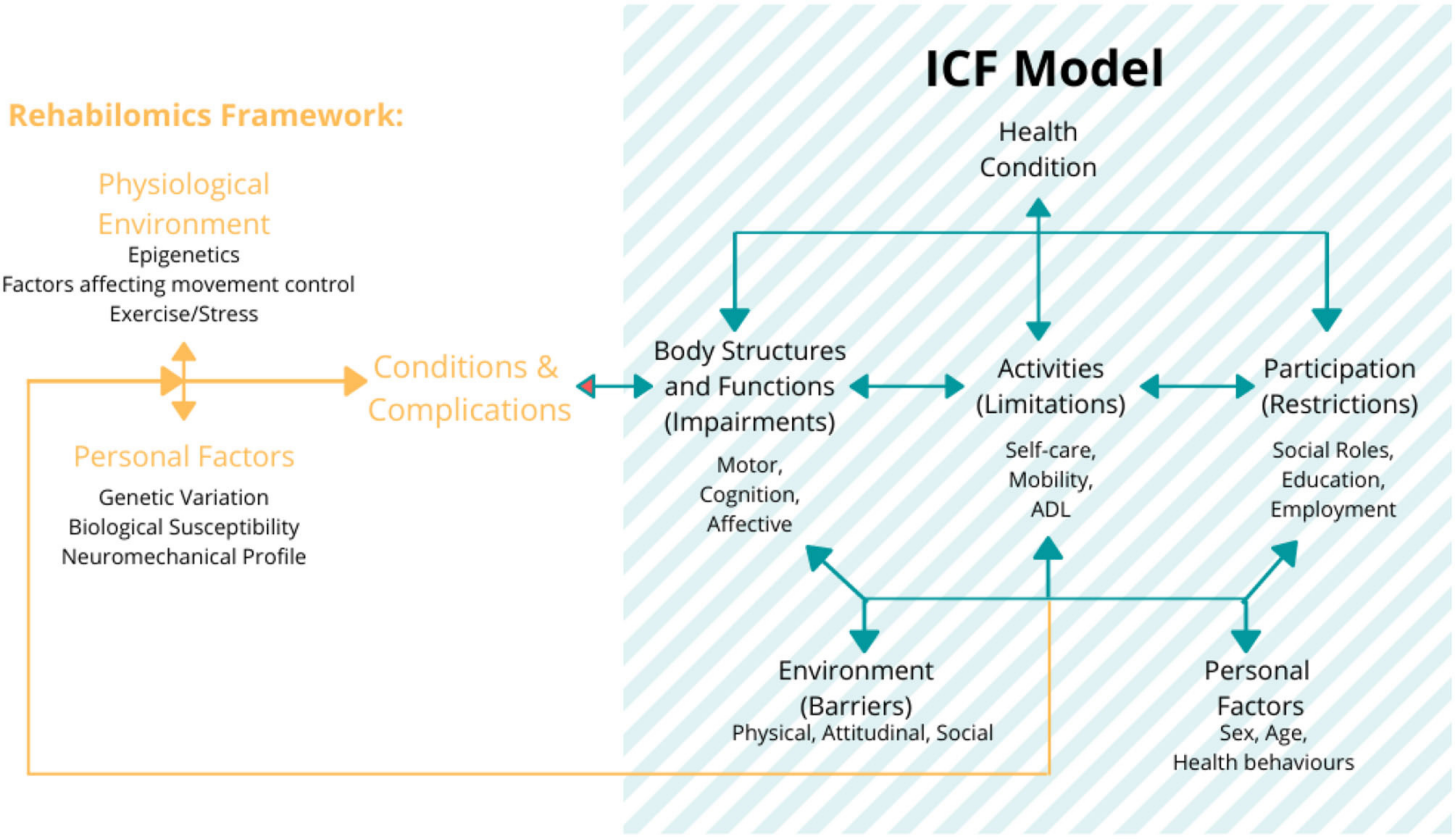
The Rehabilomics research framework uses the WHO ICF model as a foundational representation of function for the biomarker-based assessments of the brain injury response to demonstrate how these biological constructs inform the multidimensional aspects of the motor function. The figure also describes that these functional domains affect the life satisfaction and also have feedback effects on the biological impact on the health and function. Figure is open access courtesy of the National Academies of Sciences (2021) (Trang et al., 2020). Adapted from Wang et al. (2014) with permission.

In addition, the Rehabilomics approach has been directly related to the ICF framework (as shown in Stinear, 2017 and Section What Is a Biomarker and Its Relevance for Robot-Assisted Rehabilitation? Figure 4) by linking the profile of the patients (personal factors, their conditions and complications, and physiological environment) to the different dimensions of the ICF model (Figure 6). In this approach, the biomarkers could improve the stratification of the patients based on their individual biopsychosocial profiles, which could increase the statistical power of the trials to detect the intervention effects and enhance the outcomes assessment (Wagner, 2017). Thus, the consideration of such biomarkers into the ICF domains by using the Rehabilomics approach is most likely the next step in developing an integrated assessment of the robot-assisted rehabilitation treatments, optimizing clinical assessment procedures, and enhancing the effectiveness of such interventions (Do Tran et al., 2018).

## Current Gaps in the Area

**Figure 7:**
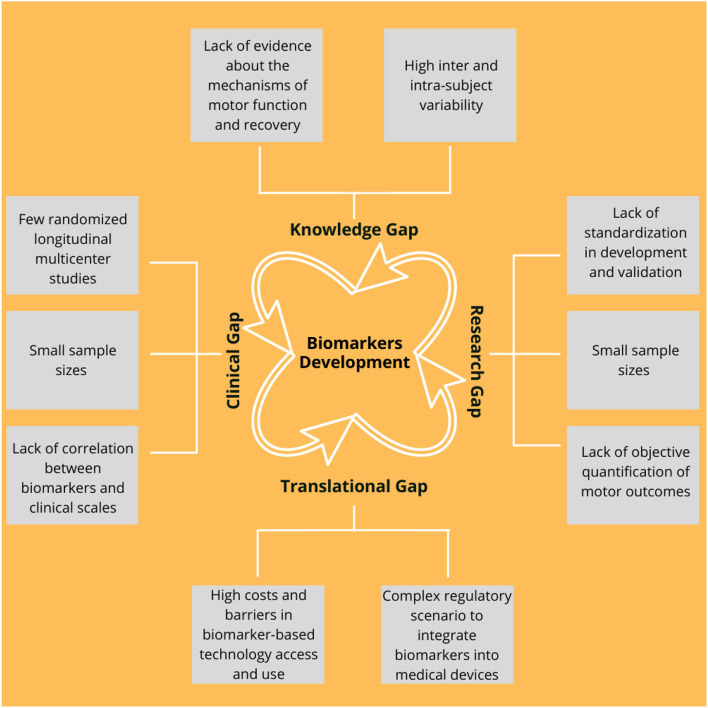
Main current gaps in the development of the biomarkers that can be grouped into the four main categories as follows: (1) Knowledge, (2) Research, (3) Translational, and (4) Clinical. A detailed description is illustrated in Table 4.

Currently, both the robotic-based interventions and the potential neurorehabilitation-based biomarkers are the presenting limitations, which are preventing their translation into the clinical practice. These can be clustered into knowledge, research, clinical, and translational gaps, which are summarized in Figure 7 and further described in Table 4.

**Table 4:** Current gaps and their implications in the translational research.

**Current gap**		**Implications in translational research**	**How to bridge the gap**
Knowledge gaps	Lack of evidence about the mechanisms of motor functions and recovery	Despite many studies have investigated the principles underlying effective neurorehabilitation, these mechanisms are still not clear (Maier et al., 2019), which hinders the translation of this knowledge into the design of biomarkers In addition, current rehabilitation practice lacks the operationalization of existing evidence from literature, leading to a gap between motor learning theory and clinical practice	Understanding and applying the processes that underline recovery mechanisms should define how patients are trained and how their assessment is quantified (i.e., how biomarkers are obtained and interpreted) Leverage on clinical practice with existing neuroscientific evidence should be applied in order to provide a functional recovery in terms of a long-term reduction of the motor impairments, instead of providing compensatory strategies (Bernhardt et al., 2017)
	There is high inter and intra-subject variability	When taking into account electrophysiological-based measures, the non-stationarity of such signals must be considered, as this could dramatically impact the stability and consequently the reliability of the computed biomarker. It is therefore pivotal to assess how signal variability intra and inter subjects and between healthy and neurological populations impacts the computation of the biomarker. As an example, muscle synergies computed from EMG signals of healthy participants show high inter subjects variability, possibly due to different motor strategies adopted by each individual and yet a synergistic description of movement at the population level emerges (Maselli et al., 2019; Scano et al., 2019). In the rehabilitation context, it may thus be difficult to discount the contribution of the individual motor strategy from the resulting pathological muscle synergies	A priority in the quest for the ideal biomarker could be to identify its robustness to intrinsic variability of the source signal. For example in the case of EEG, reproducibility of power spectrum can be assessed by making use of test-retest validations (Babiloni et al., 2020; Duan et al., 2021). These methods could therefore be exploited to investigate how electrophysiological-based biomarkers are robust to signal variability
Research gaps	Lack of standardization in development and validation	“Rehabilitation” is being used as a broad term for all types of interventions that are based in a motor therapy (Bernhardt et al., 2017). Comparison of clinical studies addressing the effects of different types of rehabilitation intervention showed that they produce similar benefits for motor recovery and outcomes, indicating that there is still no clear evidence that technological-based interventions are superior to traditional care (Stinear et al., 2020). In this context, the formulation and validation of a reliable biomarker is modality-dependent, and cannot be cross-validated across different types of therapies, leading to a lack of standardization in their computation and validation process	The introduction of the ICF model underpinned the need for a common language and reference standards in rehabilitation (Madden and Bundy, 2019). However, more standardization efforts are necessary to deal with the variability and subjectivity when measuring clinical end-points and establishing recovery biomarkers. In this line of thought, ongoing work on Rehabilomics is leading to a blueprint for characterizing biomarkers across multiple domains and interventions, ensuring their relevance to measure recovery and patient-centered outcomes (Wagner and Sowa, 2014), and their proper repeatability and reproducibility
	Lack of objective quantification of motor outcomes	Objective quantification of motor outcomes are still missing in motor rehabilitation. In particular, measurements like MCID (“the smallest difference in score in the domain of interest which patients perceive as beneficial and which would mandate a change in the patient's management” (Jaeschke et al., 1989) have been proposed, but there is no consensus regarding MCID appropriated values, which are intervention and patient-specific, and many factors can affect their computation (Beaton et al., 2002). The development of biomarkers is closely related to MCID, given that it is not enough to accurately obtain a rehabilitation-related biomarker but also to understand the clinical implications of its changes in terms of recovery, establishing an objective criteria for their relevance (Lang et al., 2008)	While there is a vast number of studies in literature identifying motor-related biomarkers, they seldom measure their outcomes in terms of MCID, or provide a criteria for interpreting the changes in the biomarkers. As part of the standardization of the development biomarkers, MCID should be included as an acceptance criteria for measuring the relevance of the biomarkers, and to allow comparison across subjects and interventions
	Small sample size	The statistical power of both clinical and research studies is strongly influenced by sample size, which leads to high variability and inconsistent results (Stinear, 2017). It has been shown that overall biomechanics studies rarely calculate sample size estimations, and they are poorly reported (Robinson et al., 2021)	Applying biomarkers to patient selection and stratification could improve rehabilitation interventions by (1) decreasing the minimal required sample size to detect relevant effects, (2) lowering recruitment time (Stinear et al., 2018) and (3) improve resolution when quantifying changes in the experimental groups
Clinical gaps	Lack of robust longitudinal multicenter studies	The design and managing of clinical trials in rehabilitation with a representative sample size poses several challenges, which vary across countries and depend on health-care systems. Factors like recruitment, patient stratification and engagement, follow-up and reporting are open issues for the deployment of large randomized multicenter clinical trials (Stinear et al., 2020). In particular, the development of potential biomarkers could lead to a further stratification of the patient population into smaller subgroups (Habermehl et al., 2018), which affects directly the sample size and the stratification criteria of the clinical study	Different strategies for improving trial quality are being proposed, which include new methods to the selection of patients, control interventions, and endpoint measures. For example, single blind, randomized, controlled (parallel-group) trials focused on defining a set of biomarkers related to long term recovery after stroke has been recently proposed (Picelli et al., 2020). Aspects like the experimental design and sample size are being addressed in fMRI-based biomarkers for multiple sclerosis (Hu et al., 2020)
	There is a lack of correlation between biomarkers and clinical scales	Clinical scales such as Fugl-Meyer Assessment (FMA) (Amano et al., 2018), Reaching Performance Scale (RPS) (Levin et al., 2004), Modified Ashworth Scale (MAS) (Harb and Kishner, 2020), Modified Rankin Scale (Quinn et al., 2009), NIH stroke scale (Lockwood, 2019), Functional Independence Measure (FIM) (Kidd et al., 1995), among others, are standard tools for clinical assessment in rehabilitation. However, attention has been called to the high variability of these scales due to different raters, level of expertise, and patient segmentation (Kanzler et al., 2020). They can also have a low resolution in terms of detecting small changes in motor function, because they do not take into account behavioral aspects, and often present “ceiling effects” (Gladstone et al., 2002) The growing development of biomarkers could help overcome these limitations (Kelly et al., 2019; Sebastian-Romagosa et al., 2020), but this exploration has not still impacted in clinical practice, which continue to guide the decision-making process depending only on traditional clinical scales (Schwarz et al., 2019), preventing from reducing sample sizes in clinical trials, and characterize motor function in a more sensitive and objective manner (Krebs et al., 2014) In particular, a systematic review focused on upper limb assessment found 49 relevant parameters in 67 state-of-the-art studies (Do Tran et al., 2018), with the aim of associating these measurements to ICF domains, and further evaluate the level of correlation of robotic-based parameters with clinical scales. The classification of kinematic parameters into these domains showed that currently no kinematic measure assesses functional performance (i.e., no parameters associated with ICF domains of Participation and Contextual Factors) Another systematic review showed 151 kinematic metrics for upper limb sensorimotor function in 255 studies (Schwarz et al., 2019). It reported that only 30 were exploring clinimetric properties, leading to a low quality of evidence, primarily attributed to the trend to focus on the development of new metrics rather that the standardization and validation of the existing ones	More efforts in adding higher resolution and quantitative measurement to existing clinical scales should be made, relying on the use of robot-based interventions. The exploration of coupling clinical scales with quantitative biomarkers is currently being exploited, with a growing number of works tackling the automatization of clinical scales through sensor data and machine learning algorithms (e.g., an automated administration of the RPS through a Kinect-based system for home rehabilitation (Scano et al., 2018), the development of prediction models combining sEMG and a set of clinical scales for hand function assessment (Baldan et al., 2021), automatization of FMA assessment (Kim et al., 2016; Julianjatsono et al., 2017; Li et al., 2017; Amano et al., 2018; Lee et al., 2018; Saes et al., 2019; Rech et al., 2020; Riahi et al., 2020)
Translational gaps	High costs and barriers in biomarker-based technology access and use	The inclusion of biomarkers to advance the efficacy of rehabilitation interventions and research is often lacking on user perspective, as poor patient and stakeholders involvement has a direct impact in the development, evaluation, and acceptance or qualification of biomarkers (Goldsack et al., 2021). In addition, the high cost and complexity of the technology necessary to deploy biomarkers adds an additional obstacle to the use of biomarkers in clinical practice, in view that it is necessary not only to acquire expensive equipment, but also to have access to high qualified personal or implement very specific training programs, often requiring staff hours that cannot be taken from patient care. Currently, biomarkers also add more time to the total rehabilitation session, which needs to be proper justified in terms of clinical benefits	The incorporation of user-centered design to biomarkers research and development could dramatically change their use in clinical settings. The importance of this approach is clear by the fact that, for example, during the development of medical devices, much effort is devoted to guarantee device usability with little training of the clinical personnel. Ease-of-use is also specifically addressed in the new medical device regulation (MDR), which has specific requirements on usability, for example regarding displays ergonomics and understandability (Wilkinson and van Boxtel, 2020). Usability should be central also for biomarker research as the adoption of user-centered design would contribute to the mitigation of the user acceptance barrier
	Complex regulatory scenario to integrate biomarkers into medical devices	The operationalization of biomarkers into clinical practice requires not only to validate their clinical relevance, but also to instrument their measurement and interpretation, and modify the regulatory framework in order to embed them into medical devices. This involves the consideration of biomarkers during the development of medical devices which will measure, compute and interpret them. In this context, the regulatory procedures relating to devices that incorporate biomarkers is complex as they can be applied to a wide range of uses and medical devices, and regulated in a different way across countries (Babrak et al., 2019) For instance, in the current regulatory framework in Europe and United States, the intended use determines whether and how the device is regulated. In particular, if the device claims to diagnose or monitor a health condition, it needs to be regulated. Especially in the case of Europe, the introduction of the new medical device regulation (MDR) focuses on the intended clinical benefits and sets high standards for guaranteeing reliable data are produced from clinical investigations (Wilkinson and van Boxtel, 2020). In addition, algorithms and software can be considered a device according to their alleged purpose, but their classification into medical devices can be difficult, requiring the intervention of regulatory bodies and long processes for certification	Several guidelines have being created in the past few years in order to establish a regulatory framework for the implementation of biomarkers (Horvath et al., 2010; Birkeland and McClure, 2015; Esteve-Pastor et al., 2019). In particular, the creation of the FDA Biomarkers Working Group has produced standards that focus on current issues related to biomarker development and regulatory acceptance (FDA-NIH Biomarker Working Group, 2016), and to create processes and policies that could help to address the challenges associated with these issues Furthermore, multidisciplinary tools for biomarkers development such as the EVIDENCE (EValuatIng connecteD sENsor teChnologiEs) checklist (Manta et al., 2021) are promoting high quality reporting in studies where the main goal is the assessment of a digital measurement. These type of guidelines are crucial for integrating clinical sciences, data management, technology development, and biostatistics into the deployment of biomarkers

## Latest Trends and Perspectives in the Field

In the previous section, some insights and future research directions have been identified. Highlights in these emerging topics are summarized in the following subsections.

### Digital Biomarkers Based on At-Home Digital Surveillance

Growing efforts in the field of mobile health are being done for improving rehabilitation therapies. On one side, the possibility of self-assessment, large-scale population screening, and continuous monitoring through mobile applications are giving rise to the development of self-paced at-home therapies by using the commonly available devices and gadgets such as smartphones and smartwatches (Zhang et al., 2020). On the other hand, the current trends on telerehabilitation (providing the rehabilitation therapies through the information and communication technologies; Cramer, 2016) have opened the possibility of providing the rehabilitation training remotely in the home of the patient or the other environments outside of the typical rehabilitation setting. The development of such remote tools for the rehabilitation management is creating a new field in the digital biomarkers (which are defined as biomarkers collected and measured by means of the digital devices; Babrak et al., 2019) related to the motor rehabilitation.

In particular, in stroke rehabilitation, wearable motor sensors are being combined with digital biomarkers to monitor the longitudinal performance of the patients (Hou et al., 2018). The state-of-the-art biomarkers such as functional range of motion (fROM) for the quantification of upper limb reaching in the 3D visualizations, convergence points (CPs) for walking analysis based on the gait parameters, and physical activity (PA) for evaluation of the energy consumption (Derungs et al., 2020) are opening the door for the exploitation of the digital biomarkers in the rehabilitation.

Initiatives such as the Parkinson's Disease Digital Biomarker DREAM Challenge (Sieberts et al., 2021) are boosting the design of the digital biomarkers-based applications for the rehabilitation. For instance, recent algorithms for the self-reported symptoms of the Parkinson's disease (Ryu et al., 2019; Zhang et al., 2020) and the biomarker-based assessments of the tremor and bradykinesia through a wrist-worn wearable (Mahadevan et al., 2020) have been published. Additionally, the exploitation of the personal devices such as the smartphones and tablets has led to the birth of the novel methods to evaluate the performance of the users. For example, tappigraphy is a non-invasive and unobtrusive method based on the screen tapping actions, which contains the important indicators of homeostasis both in the healthy and pathological conditions: for some neurological diseases, it has been already shown the efficacy of the tapping activity for the prognostic and diagnostic functions (Gindrat et al., 2015; Balerna and Ghosh, 2018; Akeret et al., 2020; Duckrow et al., 2021; Ghosh, 2021). These new type of biomarkers need not only to be clinically relevant to correctly assess the status of the patient (Manta et al., 2020), but also have to be robust enough to be recorded and interpreted under the different conditions and by the different users. Another major challenge is the requirement of the high-quality engagement of the patient necessary to obtain and deploy these biomarkers (Goldsack et al., 2021).

### Creating the Computational Neurorehabilitation Models for the Patient-Tailored Therapies

Computational models in neurorehabilitation (CMN) are encompassed by the personalized medicine and computational intelligence. CNM describes the complex human motor system in terms of the interactions between the sensorimotor activity and the behavioral outcomes of the patient by applying a computational model of the mechanisms of plasticity that are involved in recovery (Reinkensmeyer et al., 2016). It has set a framework to design the clinical experiments by simulating the rehabilitative parameters instead of using the current trial-and-error approach. This could not only allow to optimize the therapy design, but also personalize it in terms of content, timing, dosage, scheduling, etc., according to the profile of the individuals (Reinkensmeyer et al., 2016).

The concept of the patient-tailored therapies by using the computational neurorehabilitation is currently exploring the development of the new biomarkers from three main perspectives: (1) a neuroscience perspective (i.e., developing the mathematical models of the mechanisms of the activity-dependent plasticity; Reinkensmeyer et al., 2016), (2) a clinical perspective in which the clinically relevant biomarkers are being identified and used to create the algorithms for decision-making (i.e., prescribing the individualized intensities of the rehabilitation; Jeffers et al., 2018), and (3) a personalized biomechanical and sensor perspective in which the biomarkers are being used to complement the human movement analysis and wearable system design (Derungs and Amft, 2020). In particular, biomechanical simulations and motion data models are being used to create the personalized “digital twins.” This concept refers to the digital representation of the patient based on their profile health (Schwartz et al., 2020), which allows to simulate the different types of the biomarkers through this model, making the predictions and simulations of the evolution of the patient (Voigt et al., 2021) and testing and evaluating the wearable robotic systems before deploying the physical prototypes (Derungs and Amft, 2020).

### Developing an Integrated Treatment of Stroke-Induced Motor, Cognitive, and Affect-Related Deficits

Following the notion that the robot-assisted neurorehabilitation demands a highly patient-tailored process, which entails the identification of the unique needs, priorities, and recovery profile of the patient, the integration of the biomarkers belonging to the different domains (sensorimotor, cognitive-behavioral, autonomic, psychological, and psychosocial) is being undertaken (Bui and Johnson, 2018; Zariffa, 2018; Picelli et al., 2020). The idea of developing the profile of the patient that combines the relevance of the multifactorial biomarkers is a new approach that is starting to being explored, with the design of the dedicated study protocols for defining a related profile of the biomarkers of long-term recovery after stroke (Picelli et al., 2020) and the exploration of the novel biomarkers related to the other aspects of the motor function rather than sensorimotor such as alterations in the body representations (Maggio et al., 2021), eye–hand coupling assessment (Rizzo et al., 2017), quantification of visuospatial neglect (VSN) (Svaerke et al., 2019), and somatic (or cognitive-related) biomarkers (Martinez-Pernia, 2020). Additionally, the combination of the neuroimaging technologies is supporting this multifactorial exploration by combining EMG, EEG, and inertial data to obtain the rehabilitation-relevant biomarkers (Gao et al., 2018; Zhang et al., 2019; Picelli et al., 2020).

This approach could lead to the potential development of reliable one-off measures to evaluate the functionality of a single patient by developing a biomarker profile in which a reference value is present. The reference value could be a curve adjusted to the stratification of the patient with respect to the healthy population and, therefore, the value obtained from the patient could be compared against this reference, allowing to quantify the motor function in a single shot. It would be necessary to obtain and validate these reference values (or profiles) by collecting the standardized information from a large number of the patients and healthy subjects.

These multidisciplinary assessments must take into account the feasibility of their implementation in the clinical practice in which the time spent for the assessment and the level of the invasiveness and comfort for the patient are major constraints. Hence, the optimization of the calculation of biomarkers, by means of the dimensionality reduction and standardization, along with the inclusion of user-centered design principles to the process of developing new interventions and biomarkers (Markopoulos et al., 2011; Almenara et al., 2017; Wentink et al., 2019), will lead not only to the creation of the truly personalized and integrated rehabilitation technologies, but also to a significant reduction in the time spent in assessing the status of the patient.

## Conclusion

In this study, the most current relevant biomarker candidates for the rehabilitation were shortlisted and for many of them promising correlations with clinical outcomes have been found. Their use in the robot-assisted rehabilitation is at a point of the fast advancement due to the diffusion of the robotic technologies and new frameworks for multidisciplinary work such as the concept of the Rehabilomics. In particular, the development of the biomarkers based on EEG, EMG, and kinematics is a promising area in which exploratory work reported in the literature has been increasing in the recent years. Nevertheless, there are still important gaps in the area to overcome and the future studies should take into consideration more robust cross-validation protocols, addressing issues such as standardized procedures, proper sample sizes, and stratification of the patient. Further research is needed in order to identify the most informative biomarker (or set of biomarkers) to design the more optimized and patient-tailored rehabilitation therapies. This will also provide the better understanding of the prognosis and recovery and help to developing the more quantitative grounded treatment strategies to improve the recovery. This approach potentially allows a deeper understanding of the robot-assisted rehabilitation process and its interaction with the human motor control and behavioral mechanisms, boosting the development of the better human-inspired assistive technologies.

## Author Contributions

FG, MC, and MS conceived the study and reviewed the figures. FG and MS designed the figures. FG wrote the first draft of the manuscript and prepared the figures. All authors contributed to the writing of the manuscript and approved its final version.

## Funding

This study was supported by the Istituto Nazionale Assicurazione Infortuni sul Lavoro (INAIL) (Project grant number PR19-RR-P2).

## Conflict of Interest

The authors declare that the research was conducted in the absence of any commercial or financial relationships that could be construed as a potential conflict of interest.

## Publisher's Note

All claims expressed in this article are solely those of the authors and do not necessarily represent those of their affiliated organizations, or those of the publisher, the editors and the reviewers. Any product that may be evaluated in this article, or claim that may be made by its manufacturer, is not guaranteed or endorsed by the publisher.
